# Clinical factors predicting the successful discontinuation of hormone replacement therapy in patients diagnosed with primary hypothyroidism

**DOI:** 10.1371/journal.pone.0233596

**Published:** 2020-05-29

**Authors:** Kyong Yeun Jung, Hana Kim, Hoon Sung Choi, Jee Hyun An, Sun Wook Cho, Hyo Jeong Kim, Young Joo Park

**Affiliations:** 1 Department of Internal Medicine, Nowon Eulji Medical Center, Eulji University, Seoul, Republic of Korea; 2 Department of Internal Medicine, Seoul National University Hospital and Seoul National University College of Medicine, Seoul, Republic of Korea; 3 Department of Internal Medicine, Kangwon National University School of Medicine, Chuncheon, Republic of Korea; 4 Department of Internal Medicine, Korea University College of Medicine, Seoul, Republic of Korea; Boston University School of Medicine, UNITED STATES

## Abstract

**Background:**

Although reversible in some patients, primary hypothyroidism is considered a permanent condition requiring lifelong hormone therapy. This study aimed to investigate the factors predicting the successful discontinuation of levothyroxine (L–T4) therapy in patients with primary hypothyroidism.

**Methods:**

A retrospective study was performed in primary hypothyroidism patients who met inclusion criteria: patients who maintained stable L–T4 therapy for more than 1 year, following gradual dose reduction of L–T4 based on the clinical decision (L–T4 tapering); patients receiving either no L–T4 or a fixed minimum dose for more than 1 year after L–T4 tapering. Reduction in L–T4 dosage by 12.5–50 μg within 3 months was considered as L–T4 tapering. Serum free T4, TSH, and clinical symptoms were evaluated before, during and after tapering. Logistic regression and decision tree analyses were performed to predict the successful discontinuation of L–T4.

**Results:**

Among 382 patients, 22.5% and 58.4% showed successful discontinuation (T4–Discontinued) and dose reduction (T4–Reduced) of L–T4 therapy, while other did not obtained any reduction of L–T4 dose (T4–Unchanged). The median number of tapering visit was 1.0 (range, 1.0–4.0). In T4–Discontinued group, the TSH level and the positive rate of anti-thyroperoxidase at the time of L–T4 initiation were lower, the duration of L–T4 therapy was shorter, and the maintenance dose of L–T4 at the time of tapering was lower than those in the T4–Unchanged group. In ultrasonography, normal parenchyma was preserved in the T4–Discontinued group while others showed higher rates of heterogeneous or hypoechoic parenchymal changes. Among those different characteristics, the longer duration of L–T4 therapy and the higher maintenance dose of L–T4 at the time of tapering significantly predicted the failure of discontinuation of L–T4 in multivariate analysis. A decision tree showed that patients with a duration of L–T4 therapy >4.6 years had lower success rate of discontinuation.

**Conclusion:**

Shorter duration of L–T4 therapy and lower L–T4 dose at the time of tapering are the predictable factors for successful L–T4 tapering in stably maintained primary hypothyroidism patients.

## Introduction

Primary hypothyroidism is a common endocrine disorder resulting from thyroid hormone deficiency. The most common cause of hypothyroidism is autoimmune thyroiditis mediated by anti-thyroid autoantibodies [1]. The prevalence of overt hypothyroidism was reported to be 2–5% in the general population [2–4]; however, subclinical hypothyroidism is more common with a prevalence ranging from 4 to 15%, especially in iodine-sufficient areas [2, 5, 6]. Although the incidence of overt hypothyroidism is stable, the number of levothyroxine (L–T4) prescriptions has been steadily increasing worldwide over the last decade [7]. One explanation is that the increasing number of prescriptions is mostly related to subclinical hypothyroidism, which is generally detected during health screenings in asymptomatic subjects [7].

Primary hypothyroidism secondary to autoimmune thyroiditis is thought to progress to permanent hypothyroidism, due to the destruction of thyroid tissue by chronic inflammation and subsequent fibrosis [8, 9]. However, several studies have reported that more than half the number of patients recovered with iodine restriction without L–T4 replacement [10–12] and others demonstrated that 20–60% of patients remained euthyroid after L–T4 withdrawal [13–16]. In children with subclinical or overt hypothyroidism, 61% maintained an euthyroid state 3months after L–T4 withdrawal [17], and 34% required no treatment after 24 months [18]. Several factors including dietary iodine restriction [12, 19], decreased titer of antimicrosomal antibody [20], disappearance of thyrotropin-blocking antibodies [13], and recovery of thyroid responsiveness to thyroid-stimulating hormone (TSH) in a thyrotropin-releasing hormone stimulation test [14] were demonstrated as predictive factors for disease remission without L–T4 therapy. Moreover, the sonographic finding of homogenous echogenicity of the thyroid parenchyma was also suggested as a predictor for spontaneous recovery of subclinical hypothyroidism [21, 22]. The present study aimed to determine the clinical factors predicting the successful discontinuation of L–T4 therapy in primary hypothyroidism patients.

## Materials and methods

### Screening of primary hypothyroidism and eligible criteria

A retrospective chart review study was performed in three endocrinology clinics at 3 referral hospitals. The institutional review boards of Eulji Hospital (IRB no. 2018-08-012), Seoul University National Hospital (IRB no. 1708-010-873), and Korea University Hospital (IRB no. 2018AN0295) approved the study protocol.

First, we recruited a total of 11,765 patients who were diagnosed with primary hypothyroidism and had received L–T4 therapy for more than 1 year from December 2015 to December 2016. The diagnosis of primary hypothyroidism was established when the patients showed at least two elevated TSH measurements within a 3–6-month interval, with the absence of secondary causes such as thyroid surgery, radiation, or thyroid-altering medications (e.g., amiodarone, lithium, interferon). Second, patients who had been receiving stable L–T4 therapy for more than 1 year, defined as maintaining normal thyroid function without changing the L–T4 dosage, were further screened (n = 4,471). Third, we included 412 patients in whom gradual dose reduction or discontinuation of L–T4 therapy were attempted. Reduction of L–T4 dosage in a range of 12.5 to 50 μg, during the 3-month follow-up interval was considered as “gradual dose reduction” (L–T4 tapering). The fourth inclusion criterion was that the patients had to be receiving no L–T4 or a fixed minimum dose of L–T4 for ≥1 year after the tapering period (n = 398). Finally, 382 patients who had available data for TSH and free T4 measurements for at least two time points (during initiation of L–T4 tapering and at 1 year after finishing L–T4 tapering) were enrolled.

### Measurement of thyroid hormone and autoantibody levels

Serum TSH, free T4 and anti-thyroid peroxidase antibody (anti-TPO Ab) concentrations were measured via chemiluminescent immunoassay using the Abbott Architect 2000 device (Abbott Diagnostics, Lake Forest, IL, USA) at Seoul University Hospital and Advia Centaur XPT (Siemens, USA) at Nowon Eulji Medical Center, Eulji University and via radioimmunoassay using Gammapro (Seyoung-NDC Ltd., Seoul, Korea) at Korea University Hospital.

### Thyroid ultrasonography

Thyroid sonographic examinations were performed by experienced radiologists and all images were reviewed by two endocrinologists. We assessed the echogenicity and sonographic texture according to the VESINC (volume, echogenicity, sonographic texture, pseudonodular hypoechoic infiltration, nodules, and cysts) system [23]. Echogenicity criteria were described in 3 possible expressions: isoechoic, mildly hypoechoic, and hypoechoic compared to the sternocleidomastoid muscle. Sonographic texture criteria were described in 2 possible expressions: homogeneous (a regular echo pattern within the entire thyroid parenchyma with a uniform distribution of reflections), and heterogeneous (irregular echo pattern within the entire thyroid parenchyma with an uneven distribution of reflection).

### Statistical methods and decision tree modeling

Data are presented as the mean ± standard deviation. Statistical analyses via one-way analysis of variance, chi-square test, and logistic regression analysis were performed using SPSS version 18.00 (IBM Corp., Armonk, NY, USA). A P-value of <0.05 was considered significant. The decision tree model was used to find the clinical features associated with successful thyroid hormone withdrawal. The decision tree model determines the optimal cut-points of every features from the most influential to the trivial, using an information criterion such as the Gini index. To avoid overfitting problems, we applied the pruning technique, which limits the number of cases in each terminal node and the depth of the hierarchical tree. For the decision tree model, we used the Decision Tree classifier in Python with the *Scikit-learn* package.

## Results

### Clinical and biochemical characteristics of patients at the time of L–T4 initiation and during L–T4 tapering

Table 1 shows the clinical characteristics of 382 patients. The mean age was 56.1 ± 11.6 years; females were 89.8%, and the mean body mass index was 23.3 ± 3.1 kg/m^2^. At the time of initiation of L–T4 therapy, the mean free T4 and TSH values were 0.75 ± 0.30 ng/dL and 33.2 ± 40.1 μIU/mL, respectively. One hundred sixty-eight (44.0%) patients underwent anti-TPO Ab tests at diagnosis, of which 77.4% showed positive results. Before starting L–T4 tapering, the mean duration of L–T4 therapy was 7.3 ± 5.8 years and the mean dose of L–T4 was 73.2 ± 25.3 μg/day.

**Table 1:** Comparisons of clinico-biochemical characteristics among the different outcome groups after L–T4 tapering.

	Total	T4–Unchanged	T4–Reduced	T4–Discontinued	*P*
Number of patients, n (%)	382	73 (19.1)	223 (58.4)	86 (22.5)	
Age, years	56.1 ± 11.6	56.0 ± 11.6	56.6 ± 11.4	55.3 ± 12.3	0.689
Female, n (%)	343 (89.8)	59 (80.8)	203 (91.0)	81 (94.2)	0.014
Weight, kg	59.4 ± 8.8	59.1 ± 8.2	58.7 ± 8.5	61.6 ± 10.6	0.190
BMI, kg/m^2^	23.3 ± 3.1	23.2 ± 2.5	23.1 ± 3.3	24.3 ± 3.1	0.073
**At the time of L–T4 initiation**					
free T4, ng/dL					
Mean ± SD	0.75 ± 0.30	0.72 ± 0.32	0.75 ± 0.31	0.76 ± 0.28	0.809
Median [Q1-Q3]	0.76 [0.54–0.95]	0.75 [0.46–0.92]	0.77 [0.55–0.95]	0.76 [0.61–0.93]	
TSH, μIU/mL					
Mean ± SD	33.2 ± 40.1	43.7 ± 50.3	32.5 ± 37.5	25.4 ± 34.2 ^a^	0.038
Median [Q1-Q3]	16.7 [8.6–42.1]	17.6 [11.8–68.6]	16.9 [7.8–40.0]	15.5 [7.5–26.6]	
Positive TPO Ab, n (%)	130/168 (77.4)	29/41 (70.7)	82/97 (84.5)	19/30 (63.3)	0.027
**At the time of L–T4 tapering**					
Duration of L–T4 therapy, years	7.3 ± 5.8	7.7 ± 6.2	7.9 ± 5.7	5.2 ± 5.4 ^a^^,^^b^	0.002
L–T4 dose, μg/day	73.2 ± 25.3	75.0 ± 25.9	75.6 ± 24.7	64.7 ± 24.7 ^a^^,^^b^	0.003
free T4, ng/dL					
Mean ± SD	1.30 ± 0.25	1.25 ± 0.25	1.32 ± 0.25	1.30 ± 0.26	0.085
Median [Q1-Q3]	1.28 [1.11–1.43]	1.23 [1.03–1.47]	1.30 [1.14–1.41]	1.24 [1.10–1.45]	
TSH, μIU/mL					
Mean ± SD	1.5 ± 1.2	2.0 ± 1.6	1.3 ± 1.0 ^a^	1.4 ± 0.9	0.032
Median [Q1-Q3]	1.4 [0.6–2.2]	1.4 [0.6–2.8]	1.3 [0.5–1.9]	1.5 [1.1–2.1]	
Positive TPO Ab, n (%)	152/236 (64.4)	43/60 (71.7)	94/144 (65.3)	15/32 (46.9)	0.057
Number of taper, Median [range]	1.0 [1.0–4.0]	1.0 [1.0–2.0]	1.0 [1.0–4.0]	1.0 [1.0–3.0]	
Dose reduction, μg/day, Median [range]	25.0 [10.7–50.0]	25.0 [10.7–50.0]	25.0 [10.7–50.0]	25.0 [12.5–50.0]	
Follow up interval, day	97.7 ± 24.9	95.5 ± 20.5	98.2 ± 24.7	99.3 ± 31.1	0.678

BMI, body mass index; L–T4, levothyroxine; TPO Ab, thyroid peroxidase antibody

Reference ranges: free T4 0.80–1.76 ng/dl, TSH 0.55–4.78 μIU/ml, TPO Ab 0–60 IU/ml

^a^ *P* value < 0.05 vs Maintenance,

^b^ *P* value < 0.05 vs Reduction by one-way ANOVA

At the time of L–T4 tapering, the mean free T4 and TSH levels were 1.30 ± 0.25 ng/dL and 1.5 ± 1.2 μIU/mL, respectively. The median dose reduction of L–T4 was 25.0 (range, 10.7–50.0) μg/day at each visit, and the mean follow-up interval was 97.7 ± 24.9 days. During L–T4 tapering, the median number of taper was 1.0 (range, 1.0–4.0), and the mean total duration was 8.3 ± 4.0 months. Among all participants, 153 (40.1%) patients underwent serial measurement of anti-TPO Ab. Of these, 114 (74.5%) patients showed positive results at the time of L–T4 initiation, and 22 (19.3%) patients who initially tested positive for anti-TPO Ab showed negative conversion of anti-TPO Ab at the time of L–T4 tapering.

### Clinical outcomes of L–T4 tapering

Finally, 86 (22.5%) patients achieved complete discontinuation (designated “T4–Discontinued”), and 223 (58.4%) patients achieved dose reduction but not complete discontinuation of L–T4 (designated “T4–Reduced”). The other 73 (19.1%) patients did not achieve dose reduction of L–T4 (designated “T4–Unchanged”). The reasons for failure of L–T4 tapering in the T4–Unchanged group were the elevation of TSH levels over 10 μIU/mL with or without any clinical symptoms (n = 27, S3 Table) or the modest elevation of TSH levels in a range of 5−10 μIU/mL with clinical symptoms including severe fatigue, facial edema, or constipation (n = 46, S3 Table).

### Comparing characteristics according to the different clinical outcome of L–T4 tapering

Table 1 shows a comparison of the clinical characteristics and laboratory parameters among the three groups. There were no differences in age, body weight, and body mass index among groups. The proportion of females was significantly higher in the T4–Discontinued group than in the T4–Reduced or T4–Unchanged groups (94.2 vs 91.0 or 80.8%, respectively; P = 0.014). The duration of L–T4 therapy was significantly shorter (5.2 ± 5.4 vs 7.9 ± 5.7 or 7.7 ± 6.2 years; P = 0.002) and the maintenance dose of L–T4 was lower (64.7 ± 24.7 vs 75.6 ± 24.7 or 75.0 ± 25.9 μg/day; P = 0.003) in the T4–Discontinued group than in the T4–Reduced or T4–Unchanged groups. The serum TSH level at the time of L–T4 initiation was significantly lower in the T4–Discontinued group than in the T4–Unchanged group, and the serum TSH level at the time of L–T4 tapering was significantly lower in the T4–Reduced group than in the T4–Unchanged group. The serum free T4 levels were not different among the groups both at the times of L–T4 initiation and tapering. The positive rate of anti-TPO Ab (%) at the time of L–T4 initiation was lower in the T4–Discontinued group than in the T4–Reduced or T4–Unchanged groups. However, the positive rate of anti-TPO Ab (%) at the time of L–T4 tapering and the negative conversion of anti-TPO Ab did not differ among the groups.

Since previous studies have shown that successful L–T4 tapering could be achieved in a subset of patients with subclinical hypothyroidism [24, 25], we further studied the clinical outcomes of L–T4 tapering to determine if there were any differences between overt and subclinical hypothyroidism (S1 Table). Subclinical hypothyroidism was defined as serum free T4 levels within the reference range (0.8–1.8 ng/dL) and a TSH level above the upper limit of the reference range (0.5–4.8 μIU/mL), while overt hypothyroidism was defined as increased TSH level and serum free T4 levels lower than the reference range. However, there was no difference in the fractions of patients who achieved dose reduction or L–T4 discontinuation between the subclinical and overt hypothyroidism groups.

### Comparisons of thyroid sonographic findings according to the different outcome groups of L–T4 tapering

**Fig 1:**
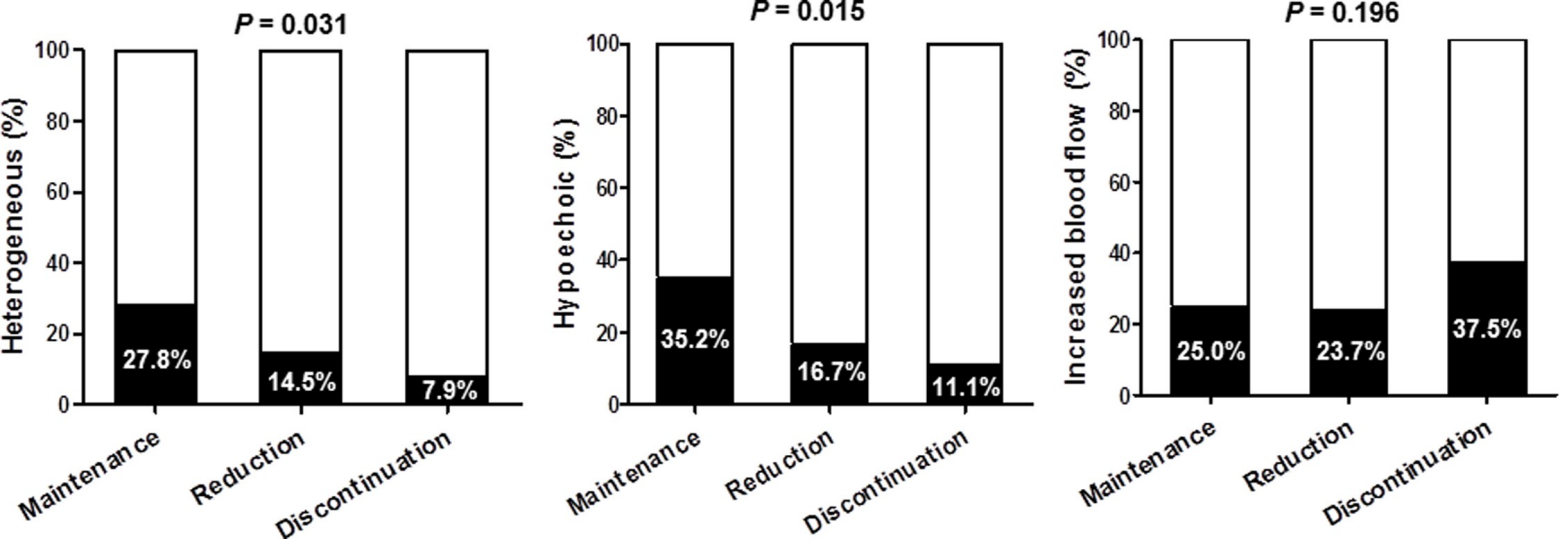
Comparisons of thyroid sonographic findings according to the different outcome groups of L–T4 tapering.

Ultrasonographic findings were analyzed among 255 (66.7% of all subjects) patients who underwent thyroid ultrasonography within 3 years prior to L–T4 tapering. Interestingly, heterogeneous or hypoechoic parenchyma was less frequently observed in the T4–Discontinued group than in the T4–Reduced or T4–Unchanged groups (Fig 1). Blood flow measured via color Doppler ultrasound showed no difference among groups. S1 Fig. shows representative images of the T4–Unchanged group (A, B) and the T4–Discontinued group (C, D).

### Predicting factors for the successful discontinuation of L–T4 therapy

To investigate the clinical factors predicting the successful discontinuation of L–T4 therapy, we first performed logistic regression analysis (Table 2). Clinical and biochemical factors which showed significant differences between the three groups in Table 1 and the ultrasonographic features were used. Among 7 factors, the duration of L–T4 therapy and the maintenance dose of L–T4 at the time of tapering significantly predicted the failure of L–T4 discontinuation in both univariate and multivariate analyses (Table 2).

**Table 2:** Predicting factor for failure to discontinuation of L–T4 therapy.

	Univariate		Multivariate	
	OR (95% CI)	P	OR (95% CI)	P
Sex (Male)	2.102 (0.796–5.553)	0.134	─	─
Duration of L–T4 therapy, years	1.113 (1.048–1.182)	0.001	1.087 (1.021–1.156)	0.009
L–T4 dose at the time of tapering, μg/day	1.019 (1.008–1.030)	0.001	1.014 (1.003–1.026)	0.017
TSH at the time of L–T4 initiation, μIU/mL	1.008 (0.999–1.017)	0.089	─	─
TSH at the time of L–T4 tapering, μIU/mL	1.081 (0.864–1.353)	0.494	─	─
USG finding (Heterogeneous)	2.586 (0.966–6.920)	0.058	─	─
USG finding (Hypoechoic)	2.240 (0.951–5.278)	0.065	─	─

OR, odds ratio; CI, confidence interval

Next, the decision tree model was used to classify the patients into two groups (success and failure groups) for the discontinuation of L–T4; this was based on age, sex, the duration and the maintenance dose of L–T4 therapy before starting L–T4 tapering, and the serum TSH level at the time of L–T4 tapering. Although the serum TSH level, the positive rate of anti-TPO Ab (%) at the time of L–T4 initiation, and the heterogeneous and hypoechoic findings of thyroid ultrasonography were significantly different between groups (Table 1 and Fig 1), these were not used because of missing data.

The optimal parameters for the pruning method were determined by the grid search for the maximum depth and the minimum number of cases at the terminal nodes using five-fold cross-validation. As a result, we selected a decision model consisting of a maximum depth of 2 and a minimum of 30 cases at each terminal node. In the decision tree, the feature importance of attributing factors was scored, and the value ranged from 0 (unused in the model) to 1 (completely predictive). Among the clinical factors used in our decision tree model, “the duration of L–T4 therapy before starting L–T4 tapering” and “serum TSH level at the time of L–T4 tapering” were the most important features, with a feature importance of 0.438 and 0.439, followed by “L–T4 doses” with feature importance values of 0.123. Age and sex, with a feature importance of 0, were not used in our model.

**Fig 2:**
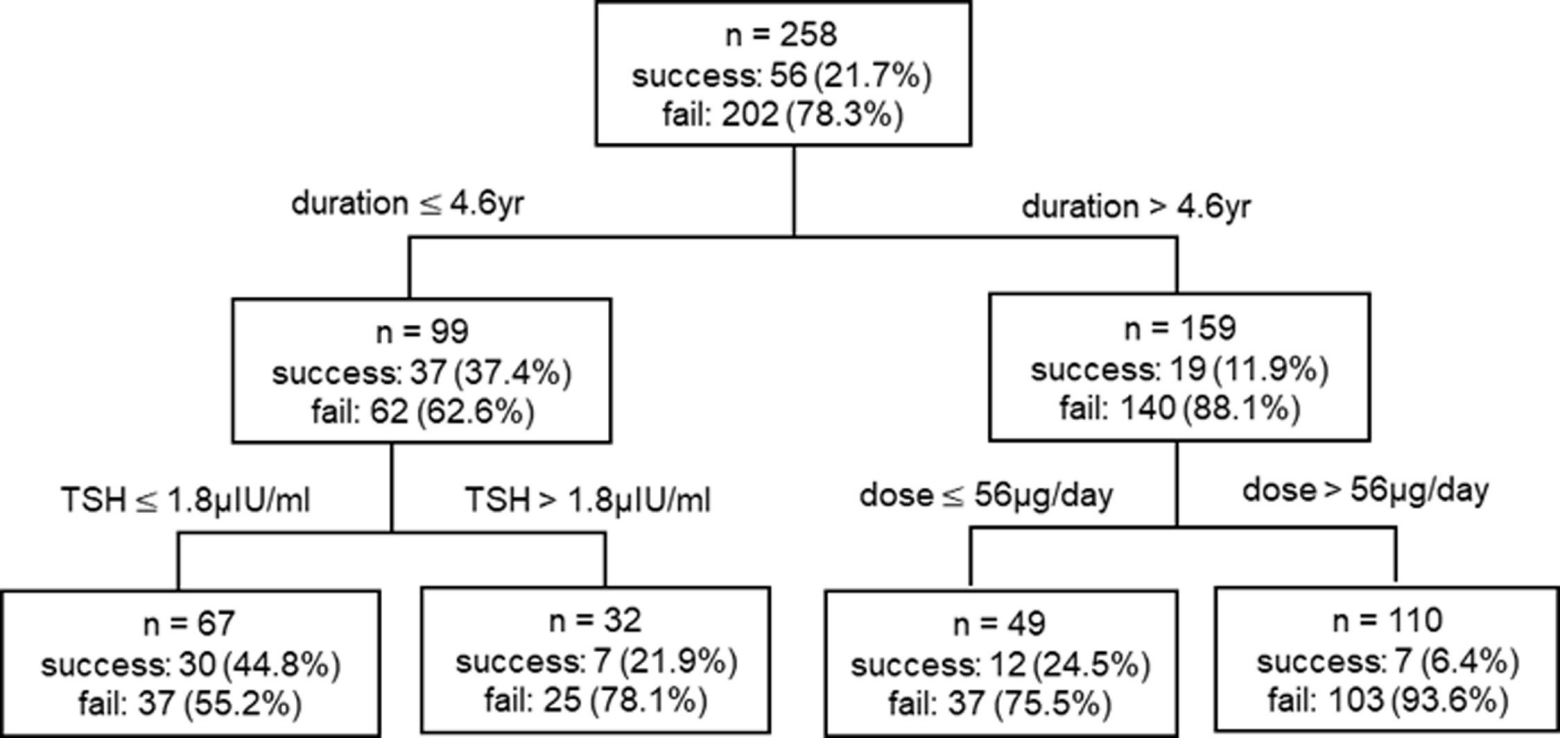
Decision tree for classifying hypothyroid patients according to the success for discontinuation of L–T4.

Fig 2 shows the decision tree model for classifying the patients into two groups (success and failure groups) according to the possibility of discontinuation of L–T4. Patients with a duration of L–T4 therapy > 4.6 years had lower success rates of discontinuation. Additionally, patients with shorter duration ≤ 4.6 years and serum TSH level ≤ 1.8 μIU/mL at the time of L–T4 tapering showed the highest success rate (44.8%). Interestingly, ROC analysis showed that the decision tree model showed higher sensitivity (0.910 vs 0.657) and lower specificity (0.210 vs 0.769) in classifying the patients into success and failure groups compared to the logistic regression model (S1 Table and S2 Fig 2). The duration and the maintenance dose of L–T4 therapy before starting L–T4 tapering showed better performance than other individual factors (S2 Table and S2 Fig).

## Discussion

Present study demonstrated that 22.5% of 382 stably maintained primary hypothyroidism patients who tried L–T4 tapering could successfully discontinue L–T4 therapy. The shorter duration of L–T4 therapy and the lower maintenance dose of L–T4 at the time of tapering was significant predicting factor for successful L–T4 discontinuation. Additionally, the absence of hypoechoic or heterogenous parenchymal changes in ultrasonography is potent predicting factor for successful L–T4 discontinuation.

Since the most common etiology of primary hypothyroidism is an autoimmune disorder which is a chronic lifelong condition, it is not clear how to delineate the optimal follow-up duration to conclude on successful L–T4 tapering, resulting in complete discontinuation. Indeed, previous studies showed 30–60% of L–T4 discontinuation among hypothyroid patients with L–T4 replacement [15–18, 26], which was higher than that obtained in the present study (22.5%). These differences can be explanted by several aspects. First, the present study was performed on relative elderly patients (diagnostic age 56.1 ± 11.6 years), while the previous studies were performed on childhood to young adolescent patients [17, 18]. Second, the present study recruited all study subjects from three referral hospitals, thus these patients had more change to review the solid diagnosis of primary hypothyroidism or the reason for receiving L–T4 replacement. Nonetheless, more than 20% of patients can completely tapering off L–T4, suggesting that the periodic reassessment of the optimal dose for L–T4 therapy would be considered. Third, the tapering speeds and the follow-up durations after L–T4 tapering were different in each study.

One of the major findings of this study was that the success rates of L–T4 tapering were similar between patients with subclinical and overt hypothyroidism. At the time of L–T4 initiation, the free T4 level was 2-fold higher and the TSH level was 0.25-fold lower in the subclinical group compared with the overt hypothyroidism group. Since subclinical hypothyroidism is generally considered as a mild or compensatory form of primary hypothyroidism [27, 28], we hypothesized that the success rates of L–T4 tapering would be higher in the subclinical group than in the overt hypothyroidism group. Indeed, the maintenance dose of L–T4 was higher among patients with overt hypothyroidism than among those with subclinical hypothyroidism. However, the present study showed that the success rates of L–T4 tapering were very similar between the subclinical and overt hypothyroidism groups, at 22.8% and 24.3%, respectively. Interestingly, when we applied the suggested clinical decision model, the proportion of patients who had serum TSH level ≤1.8 μIU/mL at the time of L–T4 tapering was higher in the overt hypothyroidism group than in the subclinical hypothyroidism group (77% vs 57%, P = 0.001), while the proportion of patients with shorter duration of L–T4 therapy (≤ 4.6 years) was similar between them. Thus, since patients with overt hypothyroidism showed more favorable clinical characteristics fitting to the decision model, the success rates of patients with overt hypothyroidism were not lower than those of patients with subclinical hypothyroidism. This finding supported the usefulness of the suggested clinical decision model.

Iodine is an essential component of thyroid hormones and excessive levels of iodine intake may exacerbate and worsen autoimmune thyroiditis [29]. The prevalence of overt and subclinical hypothyroidism increased with high iodine intake [29]. Furthermore, several studies conducted in Japan, an iodine-sufficient area, reported that about 50–60% of patients with primary hypothyroidism had shown spontaneous remission or decreased TSH level after iodine restriction [10, 11, 30]. Korea is also an iodine-sufficient region; thus, appropriate iodine restriction is important for the management of hypothyroidism [19, 31]. In the present study, patients were recommended to restrict the intake of high iodine-containing foods such as seaweed soup and laver. However, further studies are required involving the measurement of the exact amount of iodine intake.

This study showed that shorter duration of L–T4 therapy and lower L–T4 dose before starting L–T4 tapering were significant predicting factor for successful L–T4 discontinuation in multivariate analysis. Additionally, this study explored the prediction of patients who would benefit from L–T4 tapering using the decision tree model classification. Among the several machine learning algorithms, the decision tree model method has several advantages; it is easy and simple to interpret the results and it mimics the decision procedure of doctors. The decision tree method also has the flexibility of handling various types of data, such as categorical and continuous variables. Due to these advantages, the decision tree method has been widely used in medical studies. In this study, decision tree modeling showed that there was little possibility of discontinuation success in patients with longer duration (> 4.6 years), which could be applied in real practice. Although our decision tree model showed lower AUC than logistic regression model, this is caused by analysis after excluding the training set to avoid overfitting problems.

Because of the retrospective study design, the present study has several limitations. First, a subset of subclinical hypothyroidism patients started L–T4 therapy with TSH <10 uIU/ml. Although they had the medical needs for initiation of L–T4 therapy such as clinical symptoms and/or dyslipidemia, it is not clear the exact need for L–T4 therapy for them since their clinical symptoms are sometimes vague. This selection bias could affect the results. Additionally, this study had large amount of missing data especially in changes of clinical symptoms during L–T4 tapering, which made it difficult to analyze whether the process of L-T4 tapering was optimal or not. Further prospective studies are needed involving well-designed initiation of L–T4 therapy. Second, sonographic findings, an essential factor for predicting the possibility of L–T4 tapering, were not included in our decision tree model because of the large number of missing values. Consistent with previous studies [21, 22, 32], sonographic findings of heterogeneous and hypoechogenic parenchymal texture suggested a lower probability of L–T4 tapering. Furthermore, the more data a model includes, the more accurately our model could predict. Third, we could not accurately investigate iodine intake although we usually advocated the restriction of iodine-rich seaweed intake to less than one or two times per week in patients with hypothyroidism. Finally, we observed thyroid function over 1 year after discontinuation of L–T4. Therefore, some patients might have needed to restart L–T4 replacement at long term follow up. Nonetheless, the present real-world study showed that once patients start L–T4 tapering, more than 95% showed good compliance to regular follow-up for more than 1 year, suggesting a comparable need for revisiting L–T4 therapy with stable patients. However, further well-designed prospective studies including an assessment of exact iodine intake and clinical symptoms are needed to resolve clinical unmet needs of this area.

## Conclusions

Shorter duration of L-T4 therapy and the lower L–T4 dose at the time of tapering is the predictable factors for successful L–T4 tapering in stably maintained primary hypothyroidism patients. Continuous reassessment of medical need for L–T4 therapy and its proper dose may be considered for optimal treatment.

## Supporting information

S1 Fig The sonographic findings of the thyroid gland. (DOCX)

S2 Fig ROC curve of predicting factor for failure to discontinuation of L–T4 therapy. (DOCX)

S1 Table Clinical characteristics and clinical outcomes of L–T4 tapering. (DOCX)

S2 Table Predicting performance of each clinical feature and a decision tree model. (DOCX)

S3 Table Clinical and biochemical response during L–T4 tapering. (DOCX)
