# Supplementary material for: Clinical factors predicting the successful discontinuation of hormone replacement therapy in patients diagnosed with primary hypothyroidism
Source: PLoS One. 2020 May 29;15(5):e0233596. doi: 10.1371/journal.pone.0233596 (PMC7259697; doi:10.1371/journal.pone.0233596)
Supplement: S1 Fig — (DOCX) [file pone.0233596.s001.docx]

**S Fig 1.** The sonographic findings of the thyroid gland


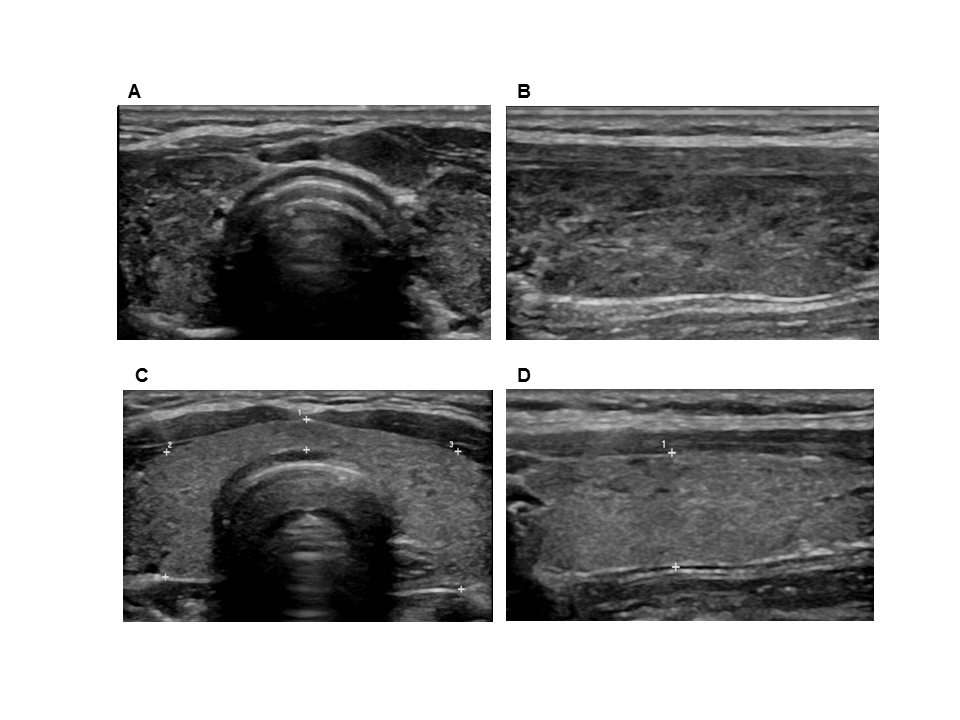


Representative images of sonographic findings of the T4–Unchanged group (A,B) and the T4–Discontinued group (C, D). Heterogeneous (A) and hypoechoic (B) were frequently found in the T4–Unchanged group, while homogeneous (C) and mild hypoechoic (D) were found in the T4–Discontinued group.
