# Supplementary figures and images for: Clinical factors predicting the successful discontinuation of hormone replacement therapy in patients diagnosed with primary hypothyroidism
Source: PLoS One. 2020 May 29;15(5):e0233596. doi: 10.1371/journal.pone.0233596 (PMC7259697; doi:10.1371/journal.pone.0233596)

**S Fig 2.** ROC curve of predicting factor for failure to discontinuation of L–T4 therapy

**
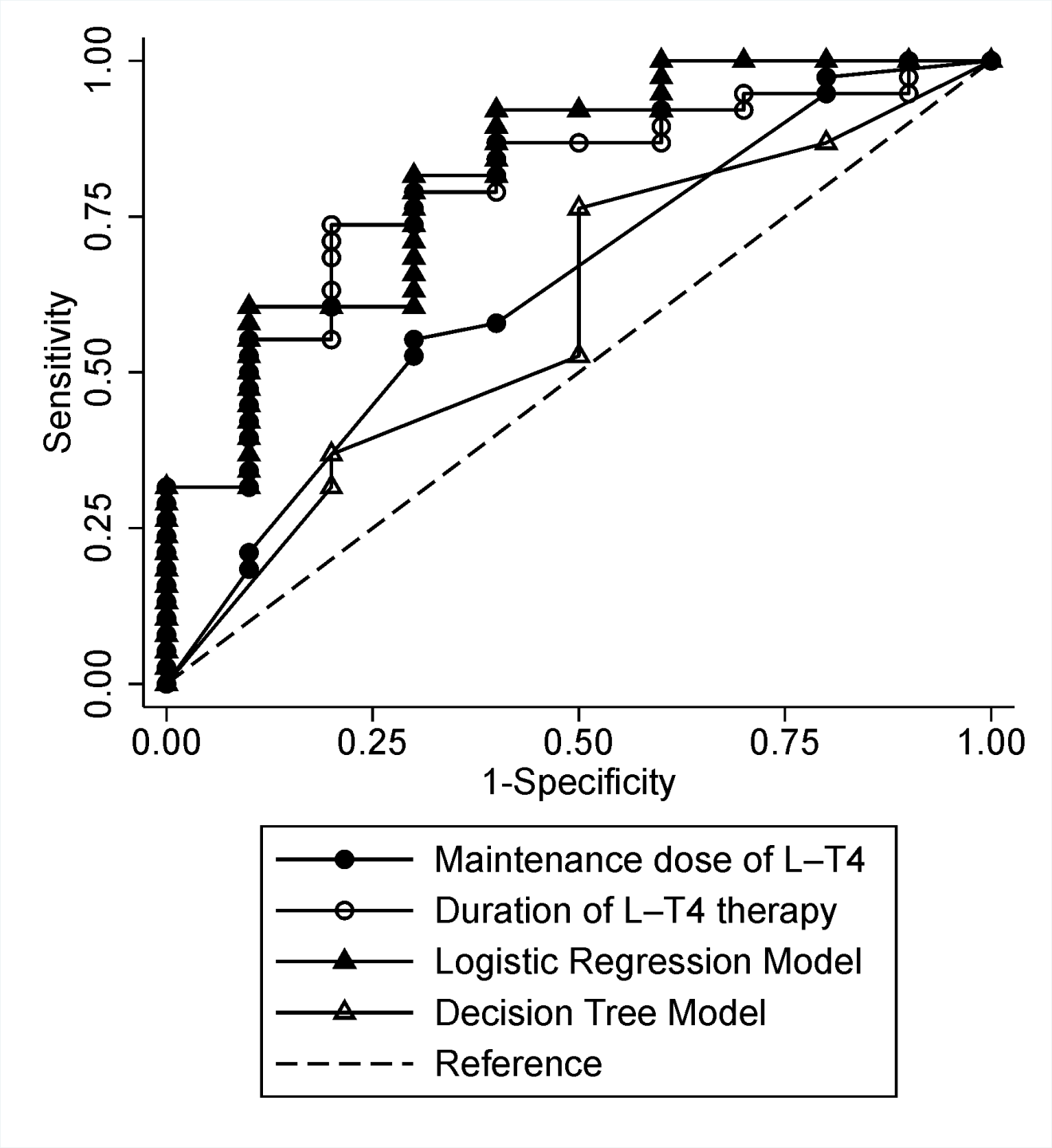
**

Supplement: S2 Fig — (DOCX) [file pone.0233596.s002.docx]
