# Supplementary material for: Clinical factors predicting the successful discontinuation of hormone replacement therapy in patients diagnosed with primary hypothyroidism
Source: PLoS One. 2020 May 29;15(5):e0233596. doi: 10.1371/journal.pone.0233596 (PMC7259697; doi:10.1371/journal.pone.0233596)
Supplement: S1 Table — (DOCX) [file pone.0233596.s003.docx]

**S Table 1.** Clinical characteristics and clinical outcomes of L–T4 tapering

| **Parameter** | **Subclinical hypothyroidism** | **Overt hypothyroidism** | ***P*** |
| --- | --- | --- | --- |
| Number of patients | 114 | 140 |  |
| Age, years | 56.2 ± 11.3 | 56.1 ± 11.6 | 0.960 |
| Female, n (%) | 95 (83.3) | 128 (91.4) | 0.050 |
| Weight, kg | 60.1 ± 10.3 | 58.7 ± 7.4 | 0.352 |
| BMI, kg/m^2^ | 23.4 ± 3.1 | 23.1 ± 2.9 | 0.552 |
| **At the time of L–T4 initiation** |  |  |  |
| free T4, ng/dL |  |  | <0.001 |
| Mean ± SD | 1.01 ± 0.16 | 0.53 ± 0.21 |  |
| Median [Q1-Q3] | 0.96 [0.89-1.08] | 0.57 [0.36-0.70] |  |
| TSH, μIU/mL |  |  | <0.001 |
| Mean ± SD | 12.7 ± 15.5 | 51.3 ± 46.7 |  |
| Median [Q1-Q3] | 9.4 [6.4-15.0] | 35.8 [16.4-76.6] |  |
| Positive TPO Ab, n (%) | 52/72 (72.2) | 70/86 (81.4) | 0.171 |
| **At the time of L–T4 tapering** | |  |  |
| Duration of L–T4 therapy, years | 5.9 ± 4.1 | 6.2 ± 4.8 | 0.575 |
| L–T4 dose, µg/day | 67.3 ± 22.9 | 78.0 ± 26.4 | 0.017 |
| free T4, ng/dL |  |  | 0.641 |
| Mean ± SD | 1.28 ± 0.22 | 1.31 ± 0.27 |  |
| Median [Q1-Q3] | 1.24 [1.12-1.43] | 1.30 [1.10-1.43] |  |
| TSH, μIU/mL |  |  | 0.199 |
| Mean ± SD | 1.7 ± 1.2 | 1.4 ± 1.1 |  |
| Median [Q1-Q3] | 1.50 [0.6-2.5] | 1.3 [0.6-1.7] |  |
| Positive TPO Ab, n (%) | 40/65 (61.5) | 60/86 (69.8) | 0.290 |
| Number of taper, Median [range] | 1.0 [1.0-3.0] | 1.0 [1.0-4.0] |  |
| Dose reduction, µg/day, Median [range] | 25.0 [10.7-50.0] | 25.0 [10.7-50.0] |  |
| Follow up interval, day | 98.4 ± 24.4 | 97.8 ± 27.3 | 0.881 |
| **Clinical outcomes of L–T4 tapering** | |  |  |
| T4–Unchanged, n (%) | 22 (19.3) | 26 (18.6) | 0.960 |
| T4–Reduced, n (%) | 66 (57.9) | 80 (57.1) |  |
| T4–Discontinued, n (%) | 26 (22.8) | 34 (24.3) |  |

BMI, body mass index; L–T4, levothyroxine; TPO Ab, thyroid peroxidase antibody

Reference ranges: free T4 0.80–1.76 ng/dl, TSH 0.55–4.78 μIU/ml, TPO Ab 0–60 IU/ml

p-value by independent *t*-test or chi-square test.
