# Supplementary material for: Clinical factors predicting the successful discontinuation of hormone replacement therapy in patients diagnosed with primary hypothyroidism
Source: PLoS One. 2020 May 29;15(5):e0233596. doi: 10.1371/journal.pone.0233596 (PMC7259697; doi:10.1371/journal.pone.0233596)
Supplement: S2 Table — (DOCX) [file pone.0233596.s004.docx]

**S Table 2.** Predicting performance of each clinical feature and a decision tree model.

|  | **Sensitivity** | **Specificity** | **AUC** |
| --- | --- | --- | --- |
| Sex (Male) | 0.115 | 0.942 | 0.528 |
| Duration of L–T4 therapy, year | 0.645 | 0.582 | 0.622 |
| L–T4 dose at the time of tapering, µg/day | 0.604 | 0.701 | 0.665 |
| TSH at the time of L–T4 initiation, μIU/mL | 0.361 | 0.820 | 0.594 |
| TSH at the time of L–T4 tapering, μIU/mL | 0.214 | 0.870 | 0.497 |
| USG finding (Heterogeneous) | 0.182 | 0.921 | 0.552 |
| USG finding (Hypoechoic) | 0.219 | 0.889 | 0.554 |
| Logistic Regression Model | 0.657 | 0.769 | 0.747 |
| Decision Tree Model | 0.910 | 0.210 | 0.603 |
