# Supplementary material for: Clinical factors predicting the successful discontinuation of hormone replacement therapy in patients diagnosed with primary hypothyroidism
Source: PLoS One. 2020 May 29;15(5):e0233596. doi: 10.1371/journal.pone.0233596 (PMC7259697; doi:10.1371/journal.pone.0233596)
Supplement: S3 Table — (DOCX) [file pone.0233596.s005.docx]

**S Table 3.** Clinical and biochemical response during L–T4 tapering

|  | **T4–Unchanged**  **(n=73)** | **T4–Reduced**  **(n=222)** | **T4–Discontinued**  **(n=86)** |
| --- | --- | --- | --- |
| Serum TSH |  |  |  |
| > 10 (μIU/mL), n | 27 | 15 | 0 |
| 5−10 (μIU/mL), n | 46 | 67 | 2 |
| Clinical symptom |  |  |  |
| missing, n | 8 | 49 | 29 |
| with progressive symptoms, n | 44 | 29 | 0 |
| without progressive symptoms, n | 21 | 144 | 57 |

Clinical symptoms were severe fatigue, facial edema, weight gain or constipation.
